# Enhancement of the Functional Performance of Cotton and Polyester Fabrics upon Treatment with Polymeric Materials Having Different Functional Groups in the Presence of Different Metal Nanoparticles

**DOI:** 10.3390/polym15143047

**Published:** 2023-07-14

**Authors:** Eman Abd El-Aziz, Menna Zayed, Amina L. Mohamed, Ahmed G. Hassabo

**Affiliations:** 1 Textile Printing, Dyeing and Finishing Department, Faculty of Applied Arts, Benha University, Benha P.O. Box 15123, Egypt; emanabdelaziz@fapa.bu.edu.eg (E.A.E.-A.); mennazaied525@gmail.com (M.Z.); 2 National Research Centre (Scopus Affiliation ID 60014618), Textile Research and Technology Institute, Pretreatment, and Finishing of Cellulose-Based Fibres Department, 33 El-Behouth St. (Former El-Tahrir Str.), Dokki, Giza P.O. Box 12622, Egypt; alo.mohamed12@hotmail.com

**Keywords:** cotton fabric, *Psidium guajava* leave extract, antibacterial, antioxidant, ultraviolet protection

## Abstract

This work examined the functional properties of three different treated fabrics, cotton, polyester, and cotton/polyester, with different polymeric materials (polyvinyl alcohol (PVA), carboxymethyl cellulose (CMC), or chitosan) in the presence and absence of two synthesized metal nanoparticles to impart and enhance fabric properties. Both metal nanoparticles (silver nanoparticle (AgNPs) and Zinc oxide nanoparticles (ZnONPs)) were synthesized using *Psidium guajava* Leaves and characterized using different techniques. The different treated fabrics were dyed with Reactive Dye (Syozol red k-3BS) and evaluated for their color strength, fastness properties, ultraviolet protection, antimicrobial activity, and mechanical properties. Results showed that treatment with polyvinyl alcohol (PVA), carboxymethyl cellulose (CMC), or chitosan enhances the functionality of all fabrics, with improved color strength, UV protection, and antimicrobial properties. Additionally, mechanical properties were slightly increased due to the creation of a thin film on the fabric surface. All dyed treated fabrics showed good ultraviolet protection and antimicrobial properties. The K/S of all treated textiles including nanoparticles and polymers was marginally greater than that of the treated materials without polymers. The UPF values demonstrate that the three investigated polymers and both metal nanoparticles enhance the fabrics’ ability to block UV radiation and shield people’s skin from its damaging effects. All treated textiles had UPF values that are higher than those of untreated textiles. Further research demonstrates that ZnONP-treated textiles exhibited greater UPF values than AgNP-treated textiles when the polymer component was present. Antibacterial examination demonstrated that treated materials had robust microbial resistance. This resistance is diminished by washing, but still prevents bacterial growth more effectively than untreated textiles.

## 1. Introduction

Currently, unique textile aids that provide high-performance textile functional finishes with exceptional high color intensity and antimicrobial activity are being developed using scientific advancements. The safety of auxiliaries is a topic that consumers are becoming more aware of and concerned about [1]. The textile industry today offers a variety of commercial auxiliary products with antibacterial qualities under various brand names on the market. The majority of these auxiliary components are constructed from man-made substances such as phenols, quaternary ammonium salts, organosilicons, and fatty acid derivatives [2,3].

Despite their tremendous effectiveness, synthetic substances are detrimental to human health. As a result, natural or safe auxiliaries are viewed as an alternative to synthetic ones for functionalizing textile materials [4,5].

**Scheme 1:**
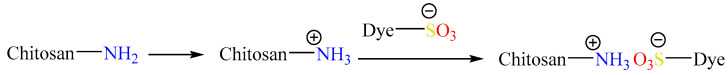
A possible mechanism for the reaction between chitosan and reactive dye.

Chitosan is a polysaccharide with anticancer properties, nontoxicity, biodegradability, and biocompatibility [6]. Chitosan was first used as a dye-deepening agent for textiles. With a few modifications, it may also be used for salt-free dyeing [7,8,9]. Chitosan causes a homogenous coating film to develop on the surface of the fiber, enhancing the fiber’s surface characteristics and reducing the repulsion between the fabric and the dyes, considerably increasing the dye absorption rate [10]. Chitosan acts as a mordant (see Scheme 1). It has an amine group (NH_2_) which quickly becomes quaternized (becomes positively charged) and therefore can attract (ionic–ionic interaction) the sulfate groups of reactive dyes (cotton dyes) and thus enhance the color shades and fixation and exhaustion of the dying process.

Carboxymethylcellulose (CMC) is often used as a water-binder and thickening ingredient in many different sectors, including liquid soaps, building materials, pharmaceutical formulations, and personal care items [11,12,13].

Multifunctional textiles have grown significantly over the past several years as a result of the growing awareness of safety and hygiene. Fabrics with nanoparticle (NP) coatings, particularly those with antibacterial capabilities, are in higher demand.

One of the most active fields of study is nanotechnology. Based on their unique qualities, such as size, distribution, and shape, nanoparticles differ from bulk forms of the same substance in terms of their physical characteristics. A nanoparticle (also known as a nanopowder, nanocluster, or nanocrystal) is a minuscule particle with at least one dimension less than 100 nm [14].

Numerous methods, including chemical reduction, microwave, and green route techniques, can be used to create silver and zinc nanoparticles [15]. However, there are several issues with using chemical approaches. As a result, the Green Route Technique of Nanoparticle Synthesis has received a lot of attention as a substitute method for creating nanoparticles due to its affordability and usage of less dangerous chemical ingredients. Due to the utilization of active phyto-compounds as a reducing and capping agent, the green biosynthesis of metal nanoparticles is a promising area of nanotechnology [16].

Depending on their UPF evaluation, UV-protective shields are divided into several categories based on international standards (excellent: above 40; very good: between 25–39; and good: between 15–24) [17,18,19,20]. UPF protection shields for 15 times the exposure duration, or 150 min, after 10 min of sustained exposure to UV sun radiation [21]. UPF ratings of 40 or 50 (40+) or higher are regarded as protective.

The purpose of the present work is to improve the dyeability of cotton, polyester, and their blended fabrics, and use fewer chemicals in the dyeing process through pre-treatment by using different polymers, like Carboxymethylcellulose (CMC), polyvinyl alcohol (PVA), or chitosan, via a surface modification to enhance the color strength, dye fixation, ultra-protection factor (UPF), and antimicrobial effect of dyed fabrics in the presence and absence of synthesized AgNPs and ZnONPs.

## 2. Experimental

### 2.1. Materials

For this study, Ghazel El-Mahala for Textile Industry Co., (El-Mahala, Egypt) provided all used fabric as follows: (a) bleached scoured cotton fabric (100%, 220 g/m^2^), (b) polyester fabric (100%; 160 g/m^2^), and (c) cotton/polyester fabric (50/50 blending ratio; 190 g/m^2^).

**Scheme 2:**
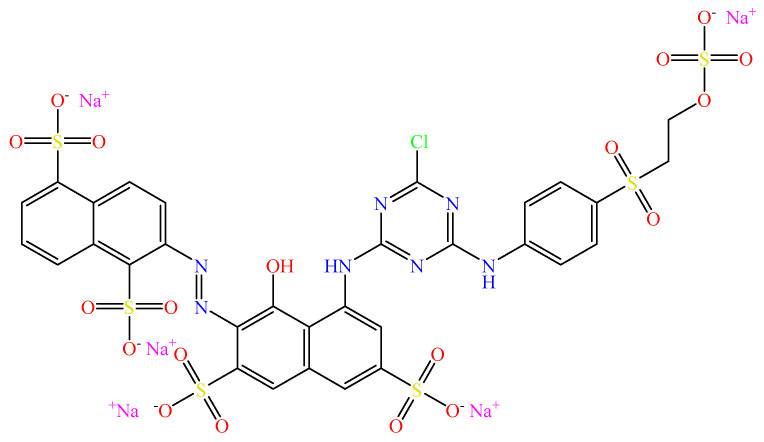
Chemical structure of Reactive Dye (Syozol red K– 3BS).

Reactive Dye (Syozol red k-3BS) was supplied by the local market in Egypt (see Scheme 2). *Psidium guajava* Leaves (*Psidium guajava* L.) were bought in Egypt at the neighborhood market. Fluka supplied citric acid, sodium hypophosphite (SHP), sodium carbonate, and acetic acid. El Nasr Pharmaceutical Chemicals Company supplied the zinc acetate. BioChemica GmbH Co., (Sauerlach, Germany), carboxymethyl cellulose (CMC, DS (0.78) at 30% of NaOH concentration) was purchased from Carl Roth GmbH Co., (Karlsruhe, Germany), polyvinyl alcohol (PVA, molecular weight 1,250,000 g/mole and degree of polymerization 1700–1800) was bought from Alpha Chemika, and chitosan low molecular weight (100,000–300,000) from ACROS Co. All of the chemicals and reagents were utilized as obtained without purification.

### 2.2. Methods

#### 2.2.1. Preparation of *Psidium guajava* Leaves (*Psidium guajava* L.) Extract

*Psidium guajava* leaves (*Psidium guajava* L.) extract was prepared as described in our previous work [22]. Natural *Psidium guajava* fresh leaves were properly cleaned and rinsed with tap water to eliminate any dust or other debris. *Psidium guajava* L. was chopped into little pieces after being thoroughly cleaned. *Psidium guajava* L. was extracted using water as a solvent to produce the extraction solution. After being heated to 100 °C for 60 min with 100 g of *Psidium guajava* L. in 1000 mL of distilled water, the extract was vacuum-filtered using Whatman filter paper No. 1. Following that, the extraction was kept at 4 °C. The filtrate was not further purified before usage.

#### 2.2.2. Synthesis of Silver Nanoparticles (AgNPs) Using *Psidium guajava* L. Extract

Silver nanoparticles (AgNPs) were synthesized as described in our previous work [23]. Aqueous AgNO_3_ (90 mL; 0.02 M) was treated with 10 mL of *Psidium guajava* L. extract for 10 min at 80 °C. To monitor the ideal conditions for the creation of silver nanoparticles (AgNPs), the pH medium was adjusted to 10. The solution’s initial color change from yellow to dark brown indicated that AgNPs were beginning to develop in the solution’s combination.

#### 2.2.3. Synthesis of Zinc Oxide Nanoparticles (ZnONPs) Using *Psidium guajava* L. Extract

Zinc oxide nanoparticles (ZnONPs) were synthesized from zinc acetate as precursors and reduced in the presence of *Psidium guajava* L. extract in water as a capping agent at pH 10 as the reported method by Zayed et al. [24] The pH of the medium was adjusted to 10 using sodium carbonate (10%) before adding 70 mL of the *Psidium guajava* L. extract in water (as a capping agent). The temperature of the solution was then increased to 70 °C, and 1 N zinc acetate in 30 mL of distilled water was gradually added while stirring the *Psidium guajava* L. extract solution for 30 min. The solution was then maintained at this temperature while being stirred for 90 min. The resulting powder was made up of Zn(OH)_2_ and Zn(CO_3_)_2_, or combinations between them such as Zn_5_(OH)_6_(CO_3_)_2_ [24], and was filtered and dried for 24 h at 90 °C. The formation of ZnONPs at the calcination stage is crucial.

#### 2.2.4. Fabric Treatment

The cotton, polyester, and cotton/polyester textiles were divided into 20 × 20 cm squares before being washed with a non-ionic detergent and air-dried. To cross-link the polymer to the fabric, a solution of 10 g/L citric acid and 5 g/L sodium hypophosphite was applied to the fabric for five minutes at 50 °C. The excess solution was wiped off, and the fabric was then air-dried. Afterwards, the fabric was immersed in polymer treatment solutions ((PVA (3%), CMC (3%) and chitosan (3%)) in the presence and absence of metal nanoparticles (AgNPs or ZnONPs).

The following steps were used to prepare an AgNPs or ZnONPs emulsion for fabric treatments in distilled water to a concentration of 10% in the presence of various polymeric ingredients (PVA, CMC, and chitosan): 100 mL of distilled water were stirred while 3 g of polymeric material was dissolved in it. For a proper dispersion of the nanoparticle inside the polymer network, 1 g of calcinated ZnONPs or 10 mL of AgNPs were introduced at 80 °C while being vigorously stirred. To ensure a perfectly homogeneous dispersion, the solution was then homogenized (in the case of utilizing ZnONPs) for 3 min at 20,000 rpm.

The finished emulsion was applied to fabrics (cotton and cotton/polyester) for 10 min, after which it was squeezed with a 100% wet pickup and dried for 5 min at 100 °C, then cured for 3 min at 140 °C. The treated fabrics were utilized for further analysis.

#### 2.2.5. Dyeing of Treated Fabrics 

Dyeing of the fabric samples was performed using a reactive dye. Dyeing parameters were subjected to (i) pH 6 (pH was adjusted using acetic acid or sodium carbonate), (ii) temperature (70 °C), and (iii) time (30 min). Finally, the dyed fabrics were rinsed with tap water and dried in an air oven at 100 °C for 5 min and cured at 140 °C for 5 min [25,26,27,28].

### 2.3. Analysis and Measurements

#### 2.3.1. Color Strength (K/S) 

Hunter Lab Ultra Scan PRO was used to assess the color strength (K/S) of untreated, treated, and dyed textiles. (Ultra-Scan PRO by Hunter Lab) (Reston, VA, USA, 2007). By using the Kubelka–Munk equation, the relative color strength (K/S) of textiles was measured and evaluated as follows [29,30,31]:

(1) $$K/S=\frac{{\left(1-R\right)}^{2}}{2R}-\frac{{\left(1-R_{o}\right)}^{2}}{2R_{o}}$$

where R_o_ is the reflectance of the white (uncolored) sample, R is the reflectance of the colored sample, S is the scattering coefficient, K is the absorption coefficient, and S is the reflectance.

#### 2.3.2. The UV Protection Factor (UPF)

The Australian/New Zealand standard (AS/NZS 4366-1996) was used to calculate the UV protection factor (UPF) for samples of treated and untreated fabrics [32]. A Cary Varian 300 UV–Vis spectrophotometer was used to measure the amount of ultraviolet transmission through the material [33].

#### 2.3.3. Mechanical Properties of the Treated Fabric

According to ASTM Test Method D1682-59T, tensile strength and elongation at break tests were performed using a tensile strength apparatus type FMCW 500 (Veb Thuringer Industrie Werk Rauenstein 11/2612 Germany) at 25 °C and 65 percent relative humidity. [34] The AATCC Test Method 66–2014 was used to test the dry crease recovery angle (CRA). [35] Using the Surface Roughness Measuring Instrument SE 1700 and ASTM Test Method D 7127–13, the fabric’s roughness was measured. [36] The ASTM test method D 1388-14e1 cantilever apparatus was used to measure stiffness. [37]

#### 2.3.4. Antibacterial Activity

According to the AATCC Test Technique, the antibacterial activity of treated textiles was quantitatively evaluated. The AATCC 100-2012 (bacterial reduction method) [38] procedure was applied to the Gram-positive bacteria (Staphylococcus aureus (ATCC 29213)), the Gram-negative bacteria (Escherichia coli (ATCC 25922)), and fungi (Candida Albicans (ATCC 10231)).

#### 2.3.5. Durability

We washed the treated cotton fabric for 10 min at 40 °C and then dried it for three minutes at 100 °C to be able to assess the treatment’s durability and the attributes it gave the cloth (for each washing cycle). The evaluated qualities were then analyzed once more following several washing cycles.

#### 2.3.6. Statistical Analysis

Three replications of each parameter were used to analyze them, and a one-way analysis of variance (ANOVA) was used to determine each parameter’s mean. Duncan’s multiple range test was used to indicate differences between samples at a 5% level (*p* 0.05).

## 3. Results and Discussion

### 3.1. Characterization of Psidium guajava L. Extract and Synthesized Nanometals

According to a variety of studies, the presence of a hydroxyl group in the molecular structure of phenolics makes them more soluble in polar solvents. Non-polar solvents have not proven as effective as polar solvents, such as ethanol and water, in the extraction process. The hydroxyl phenolic group and a polar solvent can easily interact through hydrogen bonding.

**Scheme 3:**
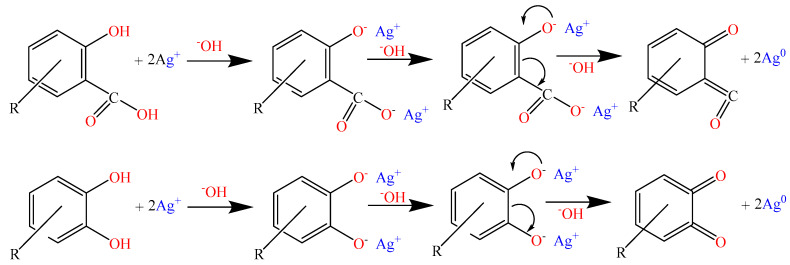
A possible suggested mechanism for the reduction of Ag^+^ by phenolic acid compound [30].

The identification of the *Psidium guajava* Leaf Extracts revealed that they are a rich source of a phenolic chemical that may be the primary factor in the bio-reduction of metal ions (M+) to metal nanoparticles (M0), as previously described. Due to their capacity to donate electrons, the phenolic compounds isolated from *Psidium guajava* leaves aid in the reduction of Ag+ and stability of Ag+ to AgNPs [22]. The possible suggested mechanism for the reduction of Ag+ by the phenolic acid compound is presented in Scheme 3 [22]. 

When metal ion solution (M+) is added to the extracted solution, the metal ions are adsorbed onto the phenolic compounds due to the electrostatic interaction between M+ ions and negatively charged alcoholate and/or carboxylate groups. This is because these phenolic compounds have hydroxyl or carboxyl groups in their chemical structure. These attraction forces encourage the creation of metal nuclei and regulate their growth while reducing the mobility of M+ [39,40].

**Figure 1:**
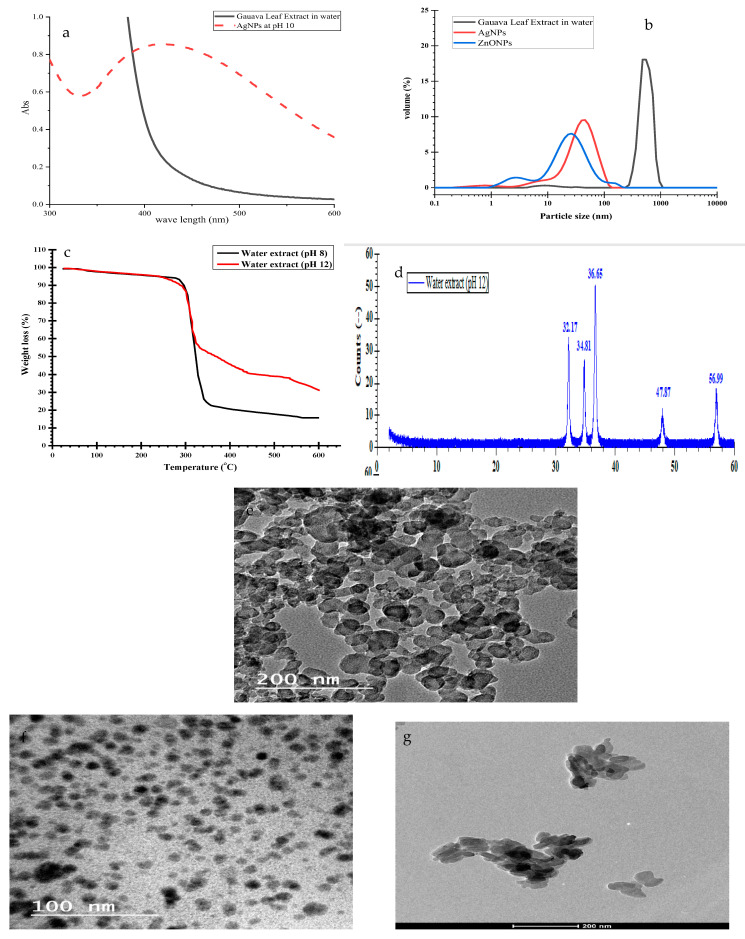
UV spectra for *Psidium guajava* L. extract in water and synthesized AgNPs [21]. (**a**) UV spectra, (**b**) particle size, (**c**) TGA, (**d**) XRD, (**e**) TEM image of *Psidium guajava* L., (**f**) TEM image of AgNPs, and (**g**) TEM image of ZnONPs.

The findings are consistent with our earlier research and have been validated. Figure 1 shows the UV–Vis spectra of silver nanoparticles that were made using *Psidium guajava* L. extract as a reducing and stabilizing agent. The colloidal solution of silver nanoparticles exhibits the distinctive peak for AgNPs in the UV–Vis spectrum at a peak absorption of 460 nm. After 10 min at 100 °C, the color changed from the original yellow to dark brown, indicating the formation of silver nanoparticles (AgNPs). Figure 1 displays the generated AgNPs and the particle size of the water-based *Psidium guajava* L. extract. Figure 1 displays TEM images of produced AgNPs and an extract of *Psidium guajava* L. in water. The TEM pictures indicate an excellent dispersion in a spherical shape. Small, spherical nanoparticles that are 5 nm in size are aggregated, according to TEM images.

As mentioned in our earlier study, guava leaves were finally employed in the production of ZnONPs at pH 12 [24]. Figure 1 shows the measured particle size of synthetic ZnO nanoparticles made with *Psidium guajava* L. extract. Figure 1 depicts the particle size of ZnONPs produced using *Psidium guajava* L. The produced ZnONPs had fewer particles. This lowering might be explained by the deposited impact of Psidium components on the ZnONPs surface.

The production of ZnONPs occurred at pH 12. Thermogravimetric studies (TGA) were used to investigate the coordinated ZnONPs for the breakdown of reduced zinc oxide with *Psidium guajava* L. Figure 1 shows water loss and capping agent breakdown at around 290 and 310 °C in the initial stage of the thermal degradation profile. However, between 310 and 320 °C, the breakdown stage came to an end. This phase also increased the weight reduction percentage from 20 to 58 percent.

A final phase terminating at 600 °C delivered a final weight reduction percent improvement of 15 to 30%. In conclusion, zinc compounds may be broken down with a favorable weight loss ratio at temperatures between 0 and 400 °C.

In their most basic form, ZnONPs are periodic structures in a 3D space. Atoms in the sample must scatter X-rays. In essence, diffractions in various directions that are typical for these atoms are produced by the dispersion of X-rays from arranged atoms. The attained peaks’ D-spacing values help identify minerals since each element has a unique set of non-overlapping D-spacings. To do this, d-spacings can be compared with common reference patterns.

For the produced ZnONPs under examination, the X-ray graphs in Figure 1 show the same high intensity and width sequence. It demonstrates that synthesized ZnONPs using *Psidium guajava* L. extract had d-Spacing values at 2 Theta positions (2θ; 32.09–32.21, 34.73–34.81, 36.53–36.69, 47.87–48.07, and 56.99–57.03) that coincided with the findings of numerous researchers in the literature in terms of values 2.7, 2.5, 2.5, 1.8, and 1.6, respectively.

Figure 1 displays TEM images of the produced ZnONPs and *Psidium guajava* L. extract on pH 12. The TEM pictures reveal a decent dispersion of spheres. According to the TEM examination, the particles of *Psidium guajava* L. have tiny scatter diameters of 80 nm. ZnONPs were also formed in nanoform when *Psidium guajava* L. extracts were present. Figure 1 illustrates how TEM images reveal that, throughout the production process, thin, spherical nanoparticles measuring 5 nm are aggregates of tiny clusters.

### 3.2. Characterization of Functionalized Fabrics

#### Metal Content (%) and the Ultraviolet Protection Factor (UPF)

The flash atomic absorption technique has been used to quantitatively quantify the total metal percent per 1 g treated textiles using synthesized metal nanoparticles with/without polymers. The findings demonstrate that the capacity of metal nanoparticles to bind to the negative charge in the polymers caused the greater binding efficiency to be recorded to treated textiles in the presence of each polymer over the pretreated fabric with citric acid and sodium hypophosphite. According to statistics, chitosan significantly increases the amount of metal absorbed into treated textiles with metal nanoparticles compared to fabrics treated with metal nanoparticles and other polymers (PVA or CMC). This is the result of charge repulsion between metals and the negatively charged molecules of polymers.

As polymers and metal nanoparticles increase this feature, further research on the treated fabric should measure the Ultraviolet protection factor to ascertain how well the treated fabric can block out ultraviolet waves.

As a result, Table 1 contains the UPF values of untreated and treated textiles with synthesized metal nanoparticles in the presence or absence of various polymer compounds. The UPF values show that all treated textiles have UPF values that are greater than those of untreated fabrics, proving that the three examined polymers and both metal nanoparticles improve the fabrics’ capacity to block UV radiation and protect people’s skin from its damaging effects. Further findings show that textiles treated with ZnONPs in the presence of the polymer compound had higher UPF values than fabrics treated with AgNPs.

According to the research, treated textiles should have UPF levels for UV protection of at least 40 to 50 or higher [20]. As a result, even after washing, all treated textiles associated with various formulations have good UV protection.

The coating’s washing endurance was demonstrated after ten washing cycles, and it was discovered that a sizable proportion of the polymers and nanometals used in the coating were still present. This highlighted the potential application of these components in protective apparel. 

By adding the metal nanoparticles to the polymer formulation, a significant increase in the K/S and UPF values was observed (*p* < 0.05).

### 3.3. Characterization of Dyed Fabrics

#### 3.3.1. Color Performance and Fastness Properties

To evaluate the efficiency of polymer and metal nanoparticles treatment on the dyed textiles, the K/S of the treated fabrics using synthesized metal nanoparticles with/without polymers was measured and recorded in Table 2. The data show the following common features: (a) the K/S of all fibers treated with nanoparticles is generally higher than that of untreated fibers. (b) The K/S of all fibers treated with nanoparticles in the presence of polymers (PVA, CMC, or chitosan) is slightly higher than that of treated fabrics without polymers. 

The color fastness of the dyed fabrics was evaluated and is reported in Table 2. The washing fastness rating of the treated dyed fabric was excellent for both fading and staining with ratings ranging from 4 to 5, while those of untreated fabrics ranged from moderate to good for staining and for fading (2–4). All the dyed treated fabrics presented very good rubbing fastness (rating 4–5), indicating good diffusion and penetration of the dye into fiber substrates. Nevertheless, the good light and perspiration fastness of all dyed fabrics was noticed. 

#### 3.3.2. Antimicrobial Properties

The main goal of the antimicrobial uses of biomaterials and metal nanoparticles is to reduce the effects of biofouling and bacterial colonization with the added benefit of eliminating the need for medications that could lead to bacterial resistance. Due to improvements against surgical complications and healthcare-associated infections, which are primarily categorized into physical or mechanical, electrostatic, and chemical divisions, these metal nanoparticles’ inherent anti-microbial and anti-fouling properties have been establishing pillars of success in the biomedical domain. Skin abnormalities, especially skin injury, have an urgent need for application-based usage of polymeric and metal nanoparticles. Skin, which is prone to infections, aids bacterial infections in spreading harm, while also focusing on internal organs and the tissues around them. The content of unsaturated fatty acids in the cell membrane changes as a result of NPs coming into contact with bacterial cells, changing the fluidity of the membrane. When bacteria are exposed to NPs, they modify the content of unsaturated fatty acids, which modifies the fluidity of the membrane and prevents NPs from entering the cell. 

Microbial growth rises with increased moisture and textile washing and peaks at neutral pH (7–8) [41]. All bacteria, except for phototropic species, can grow well in darkness. They are UV-sensitive, and exposure to light can cause pigment to form and result in colorful fabric stains [42].

Microorganisms have a cell wall that is only partially permeable to the integrity of the cellular substance. Cell damage and cell membrane rupture are the results of bactericidal agents. Bactericidal substances can stop bacteria from growing by blocking the formation of cell walls, cytoplasmic membrane permeability, physical and chemical protein and nucleic acids, enzyme activity, and protein and nucleic acid synthesis, among other effects.

Furthermore, in both dry and moist environments, metal ions can kill bacteria by strangling them. [43] The protein is bound to both the internal and external bacterial membranes by the extremely physiologically active silver and zinc ions, which prevents respiration and cell division. Products made of silver and zinc are resistant to bacteria, but they do not work against other species or fungus, mold, or mildew.

Treatment substrates were submitted for evaluation of their antimicrobial activity using the reduction method (as a quantitative method) against Escherichia coli (E. Coli; ATCC 25922), Staphylococcus aureus (S. Aureus; ATCC 29213), and Candida albicans (C. Albicans; ATCC 10231), a fungus. The results are shown in Table 3.

Additionally, treated textiles are more effective against Gram-positive bacteria than Gram-negative bacteria, which is explained by the fact that the two strains of bacteria under study have different cell wall structures.

The polyphenolic chemicals’ suppression of microbial RNA and DNA can be compatible with their antibacterial effect. It is also possible to depolarize the cytoplasmic membrane of microbes. These substances also have antifungal properties because they prevent ergosterol, the primary element of fungi’s cell membrane, from functioning.

The antibacterial activity of both untreated and treated fabric substrates against Escherichia coli, Staphylococcus aureus, and Candida albicans was assessed in the presence and absence of metal nanoparticles using the counting procedure before and after 10 washing cycles. The proportion of antibacterial decrease in each fabric is shown in Table 3. In untreated samples, there is no suppression of the decline %. The administered samples exhibited antimicrobial tolerance.

The antimicrobial findings for treated fabrics showed that, compared to Gram-positive bacteria, Gram-negative bacteria showed less sensitivity to fabrics treated with chitosan than with the other two polymers (PVA and CMC), whereas fungal strains showed a significant susceptibility. This was mainly related to changes in the makeup of each cell wall. Gram-positive bacteria have a single cell membrane that is surrounded by a thick, porous cell wall that allows certain bioactive components to pass through it. Preferably, Gram-negative bacteria have three different layers that protect certain bioactive components [44,45,46].

In the coating process, there is an effective contact between both metal nanoparticles and the bacteria cells. Chitosan is a polymer made of amino and hydroxyl groups that effectively distributes and stabilizes metal nanoparticles in solutions and on fabric surfaces, reducing the ability of germs to adhere to individual fabric surfaces. The percentage of decrease in bacteria for treated textiles exhibits the same behavior when tested against bacteria and fungi. 

Most strategies (e.g., nanoparticle coating) to achieve antibacterial activity in fabric or cellulose-based fibers explicitly show the application of techniques (for example, nanoencapsulation). In contrast, without any physical or chemical treatment before the fabric surface, the loosely entrapped antibacterial agents generally start losing significant antibacterial properties after subsequent washing. 

The number of bacteria on treated clothes after various washing cycles was examined. Up to 10 washing cycles, the amount of microbial resistance decreased, and every additional increase in washing cycles caused a modest drop in the number of bacteria on treated materials. These findings proved that treated materials have strong microbial resistance, which is diminished by washing, but still inhibits bacterial growth more than untreated fabrics. This is additional proof that these treated textiles would produce well in the medical industry.

As shown in Table 3, the control fabric lacked antibacterial activity, but when each polymer and various nanoparticles were added to the textiles, the antibacterial activity against the specified bacteria dramatically enhanced (*p* 0.05).

#### 3.3.3. Mechanical Properties

Before and after polymer treatment in the presence and absence of metal nanoparticles, the treated textiles’ tensile strength, break elongation, air permeability, roughness, and crease recovery angle were monitored. The results of the analyses are shown in Table 4.

The information in Table 4 makes it evident that the treated textiles’ physico-mechanical characteristics were a top priority. Following treatment, as surface roughness, tensile strength, and elongation at break decrease, the bending length and crease recovery angle (CRA) rises considerably. This shows that the polymers under research were thoroughly absorbed into the microstructure of the textiles, and as a result, a thin film developed on the fabric surface and was determined to be in charge of these modifications.

It was also important to underline the importance of pre-crosslinking fabric using citric acid catalyzed by sodium hypophosphite (SHP) as the dominating agent in determining the effectiveness of the aforementioned qualities. During this pre-treatment, covalent crosslinking links between nearby cellulose chains would be formed, giving the structure stiffness. The fabrics will begin to disintegrate chemically as a result of the interaction with citric acid.

The coated film, on the other hand, can fill in holes on the surface of textiles with less roughness. The significant increase in the crease recovery angle was probably caused by the development of a dense network in the fabric’s structure, which was tightly connected by covalent chemical bonds between the cellulose chain and the polymer components with carboxyl or hydroxyl groups.

## 4. Conclusions

During this work, three different fabrics, namely cotton, polyester, and cotton/polyester, were treated with three polymeric materials with different functional groups (PVA (OH group), CMC (COOH group), and chitosan (NH_2_ group) in the presence and absence of two synthesized metal nanoparticles (AgNPs and ZnONPs), and the the treated fabrics were then dyed and evaluated for their additional functional properties. Both metal nanoparticles (AgNPs and ZnONPs) were synthesized using *Psidium guajava* Leaves and characterized using different techniques, and the results provide the synthesis of both metal nanoparticles with smaller sizes and spherical- and rod-shaped AgNPs and ZnONPs, respectively. 

Different treated fabrics were dyed with Reactive Dye (Syozol red k-3BS) and then evaluated for their color strength, fastness properties, ultraviolet protection, antimicrobial activity, and mechanical properties. From all different analysis techniques, it is clear that treatment with all polymeric materials (PVA, CMC, or chitosan) enhances the functionality of all fabrics. Color strength, ultraviolet protection, and antimicrobial properties were improved and provide excellent value compared to untreated fabrics. In addition, mechanical properties were slightly increased upon treatment as a result of creating a thin film on the fabric surface which enhanced the tensile strength and other mechanical properties. Furthermore, all dyed treated fabrics showed good ultraviolet protection and antimicrobial properties. 

## Figures and Tables

**Table 1:** Metals content (%), color intensity (K/S), and Ultraviolet protection factor (UPF) for treated fabrics with/without polymers.

Fabric	Polymer Conc. (10 g/L)	MNPs (10%)	Metal Content (%/g Fabric)	K/S	UPF after Dyeing	
					Before Washing	After Washing
Cotton	Blank		0	6.23 ± 0.18	0.2 ± 0.01	0.2 ± 0.01
	PVA	Without	0	7.21 ± 0.217	3.3 ± 0.173	2.1 ± 0.11
		AgNPs	0.11 ± 0.006	7.65 ± 0.381	28.23 ± 1.478	22.13 ± 1.159
		ZnONPs	0.24 ± 0.013	7.42 ± 0.18	54.23 ± 2.839	45.32 ± 2.373
	Chitosan	Without	0	7.95 ± 0.354	5.42 ± 0.284	4.11 ± 0.215
		AgNPs	0.31 ± 0.016	8.35 ± 0.308	41.31 ± 2.163	43.21 ± 2.262
		ZnONPs	0.44 ± 0.023	8.31 ± 0.163	59.2 ± 3.1	45.3 ± 2.372
	CMC	Without	0	7.58 ± 3.145	3.25 ± 0.17	2.13 ± 0.112
		AgNPs	0.24 ± 0.013	7.78 ± 3.024	38.11 ± 1.995	36.53 ± 1.913
		ZnONPs	0.23 ± 0.012	7.98 ± 0.649	52.1 ± 2.728	44.31 ± 2.32
Polyester	Blank		0	1.21 ± 0.593	0.1 ± 0.005	0.1 ± 0.005
	PVA	Without	0	1.99 ± 0.688	2.54 ± 0.133	1.62 ± 0.085
		AgNPs	0.07 ± 0.004	2.8 ± 0.853	10.23 ± 0.536	8.21 ± 0.43
		ZnONPs	0.15 ± 0.008	3.44 ± 0.781	32.14 ± 1.683	27.41 ± 1.435
	Chitosan	Without	0	4.47 ± 1.331	4.17 ± 0.218	3.16 ± 0.165
		AgNPs	0.26 ± 0.014	5.53 ± 1.377	25.31 ± 1.325	22.63 ± 1.185
		ZnONPs	0.37 ± 0.02	6.38 ± 0.56	12.42 ± 0.65	9.63 ± 0.504
	CMC	Without	0	3.23 ± 0.602	2.51 ± 0.131	1.64 ± 0.086
		AgNPs	0.23 ± 0.012	3.76 ± 0.299	22.75 ± 1.191	20.68 ± 1.083
		ZnONPs	0.22 ± 0.012	4.59 ± 0.295	8.31 ± 0.435	5.42 ± 0.284
Cotton/Polyester	Blank		0	5.23 ± 0.352	0.17 ± 0.009	0.17 ± 0.009
	PVA	Without	0	4.6 ± 0.426	3.27 ± 0.171	2.08 ± 0.109
		AgNPs	0.1 ± 0.005	5.22 ± 0.617	21.54 ± 1.128	16.99 ± 0.89
		ZnONPs	0.22 ± 0.012	5.43 ± 0.468	48.37 ± 2.533	40.73 ± 2.133
	Chitosan	Without	0	6.21 ± 0.836	5.37 ± 0.281	4.07 ± 0.213
		AgNPs	0.32 ± 0.017	6.94 ± 0.841	37.31 ± 1.954	36.87 ± 1.931
		ZnONPs	0.46 ± 0.024	7.34 ± 0.361	40.11 ± 2.1	30.76 ± 1.611
	CMC	Without	0	5.4 ± 0.416	3.22 ± 0.169	2.11 ± 0.11
		AgNPs	0.26 ± 0.014	5.77 ± 0.416	34.08 ± 1.784	32.04 ± 1.678
		ZnONPs	0.25 ± 0.013	6.28 ± 0.255	33.83 ± 1.771	27.85 ± 1.458

**Table 2:** Color intensity (K/S) and fastness properties for treated dyed fabrics with/without polymers.

Fabric	Polym+B1:M21r Conc. (10 g/L)	MNPs (10%)	K/S	Fastness Properties								
				Washing		Rubbing		Perspiration				Light
								Acidic		Alkaline		
				Alt.	St	Dry	wet	Alt.	St.	Alt.	St.	
Cotton	Blank		6.23	4	4	4	4	4	4	4	4	5
	PVA	without	7.21	4–5	4	4–5	4–5	4–5	4–5	4–5	4	7
		AgNPs	7.65	4	4	4	4–5	4–5	4–5	4–5	4–5	6–7
		ZnONPs	7.42	4	4	4	4	4	4	4	4	7
	Chitosan	without	7.95	4	4	4	4–5	4–5	4–5	4–5	4–5	6
		AgNPs	8.35	4	4–5	4–5	4–5	4–5	4–5	4–5	4–5	7
		ZnONPs	8.31	4	4	4	4	4	4	4	4	7
	CMC	without	7.58	4	4–5	4–5	4–5	4	4–5	4–5	4–5	6
		AgNPs	7.78	4	4–5	4–5	4–5	4	4	4–6	4	6–7
		ZnONPs	7.98	4	4	4	4	4	4	4	4	6
Polyester	Blank		1.21	2–3	2	2–3	2	2	2	2	2	3
	PVA	without	1.99	4	4	4	4	4	4	4	4	5
		AgNPs	2.8	4–5	4	4–5	4–5	3–4	3–4	4–5	4	5
		ZnONPs	3.44	4	3–4	3–4	4–5	4	4–5	3–4	3–4	5
	Chitosan	without	4.47	4	4–5	3–4	3–4	4	4–5	3–4	3–4	5
		AgNPs	5.53	4	4–5	4–5	4–5	4	4–5	4–5	4–5	5–6
		ZnONPs	6.38	4	4	4	4	4	4	4	4	5–6
	CMC	without	3.23	4	4–5	3–4	3–4	4	4–5	3–4	3–4	5
		AgNPs	3.76	4–5	4	3–4	3–4	4–5	4	4–5	4	6
		ZnONPs	4.59	4	4	4	3–4	3–4	3–4	3–4	4	6
Cotton/Polyester	Blank		5.23	3–4	3–4	3–4	3–4	3–4	3–4	3–4	3–4	5
	PVA	without	4.6	4–5	4	4–5	4–5	4	4–5	4–5	4–5	6
		AgNPs	5.22	4–5	4	4–5	4–5	4	4–5	4–5	4–5	6–7
		ZnONPs	5.43	4	4	4	4	4	4	4	4	6–7
	Chitosan	without	6.21	4–5	4–5	4–5	4–5	4–5	4–5	4–5	4–5	5
		AgNPs	6.94	4	4–5	4–5	4	4–5	4	4–5	4	6–7
		ZnONPs	7.34	4	4	4	4	4	4	4	4	6–7
	CMC	without	5.4	4	4	4	4	4	4	4	4	6
		AgNPs	5.77	4	4	4	4–5	4	4–5	4–5	4	6–7
		ZnONPs	6.28	4	4	4	4	4	4	4	4	6–7

**Table 3:** Microbial reduction % for antibacterial activity of dyed fabrics treated with different polymers in the presence and absence of nano metals.

Fabric	Polymer Conc. (10 g/L)	MNPs (10%)	Microbial Reduction %					
			*E. coli* (ATCC 25922)		*S. aureus* (ATCC 29213)		*C. albicans* (ATCC 10231)	
			Before Washing	After Washing	Before Washing	After Washing	Before Washing	After Washing
Cotton	Blank		0	0	0	0	0	0
	PVA	Without	41.13 ± 1.779	38.11 ± 1.649	50.99 ± 2.206	42.04 ± 1.819	33.18 ± 1.435	30.64 ± 1.325
		AgNPs	74.86 ± 3.238	65.54 ± 2.835	87.7 ± 3.794	72.31 ± 3.128	57.07 ± 2.469	52.69 ± 2.279
		ZnONPs	80.06 ± 3.463	72.19 ± 3.123	96.6 ± 4.179	79.64 ± 3.445	62.86 ± 2.719	58.04 ± 2.511
	Chitosan	Without	55.73 ± 2.411	50.94 ± 2.204	62.24 ± 2.693	55.82 ± 2.415	47.95 ± 2.074	45.79 ± 1.981
		AgNPs	90.28 ± 3.906	82.52 ± 3.57	100.83 ± 4.362	90.42 ± 3.912	77.67 ± 3.36	74.17 ± 3.209
		ZnONPs	93.64 ± 4.051	85.6 ± 3.703	104.59 ± 4.525	93.79 ± 4.057	80.57 ± 3.485	76.94 ± 3.328
	CMC	Without	50.6 ± 2.189	46.09 ± 1.994	57.71 ± 2.497	50.75 ± 2.195	42.81 ± 1.852	40.6 ± 1.756
		AgNPs	71.86 ± 3.109	65.45 ± 2.831	81.95 ± 3.545	72.06 ± 3.117	60.8 ± 2.63	57.65 ± 2.494
		ZnONPs	73.6 ± 3.184	67.04 ± 2.9	83.93 ± 3.631	73.81 ± 3.193	62.27 ± 2.694	59.05 ± 2.555
Polyester	Blank		0	0	0	0	0	0
	PVA	Without	50.99 ± 2.206	46.17 ± 1.997	57.52 ± 2.488	51.02 ± 2.207	43.39 ± 1.877	41.27 ± 1.785
		AgNPs	72.41 ± 3.132	65.56 ± 2.836	81.68 ± 3.533	72.44 ± 3.134	61.61 ± 2.665	58.6 ± 2.535
		ZnONPs	81.4 ± 3.521	73.71 ± 3.189	91.83 ± 3.973	81.44 ± 3.523	69.26 ± 2.996	65.88 ± 2.85
	Chitosan	Without	70.52 ± 3.051	64.09 ± 2.773	74.51 ± 3.223	71.72 ± 3.103	69.45 ± 3.004	68.84 ± 2.978
		AgNPs	86.03 ± 3.722	78.19 ± 3.382	90.9 ± 3.932	87.49 ± 3.785	84.73 ± 3.665	83.98 ± 3.633
		ZnONPs	89.45 ± 3.87	81.29 ± 3.517	94.51 ± 4.089	90.97 ± 3.935	88.09 ± 3.811	87.31 ± 3.777
	CMC	Without	60.75 ± 2.628	55.13 ± 2.385	66.02 ± 2.856	61.37 ± 2.655	56.42 ± 2.441	55.05 ± 2.381
		AgNPs	74.12 ± 3.206	67.26 ± 2.91	80.54 ± 3.484	74.87 ± 3.239	68.83 ± 2.978	67.17 ± 2.906
		ZnONPs	77.06 ± 3.334	69.93 ± 3.025	83.73 ± 3.622	77.84 ± 3.367	71.56 ± 3.096	69.83 ± 3.021
Cotton/Polyester	Blank		0	0	0	0	0	0
	PVA	Without	46.06 ± 1.993	42.14 ± 1.823	54.26 ± 2.347	46.53 ± 2.013	38.28 ± 1.656	35.95 ± 1.555
		AgNPs	56.2 ± 2.431	51.41 ± 2.224	66.19 ± 2.863	56.76 ± 2.455	46.71 ± 2.021	43.86 ± 1.897
		ZnONPs	58.42 ± 2.527	53.45 ± 2.312	68.82 ± 2.977	59.02 ± 2.553	48.56 ± 2.101	45.6 ± 1.973
	Chitosan	Without	63.12 ± 2.731	57.52 ± 2.488	68.38 ± 2.958	63.77 ± 2.759	58.7 ± 2.539	57.31 ± 2.479
		AgNPs	77.01 ± 3.331	70.17 ± 3.036	83.42 ± 3.609	77.8 ± 3.366	71.61 ± 3.098	69.92 ± 3.025
		ZnONPs	80.06 ± 3.463	72.95 ± 3.156	86.73 ± 3.752	80.88 ± 3.499	74.45 ± 3.221	72.69 ± 3.145
	CMC	Without	55.68 ± 2.409	50.61 ± 2.189	61.86 ± 2.676	56.06 ± 2.425	49.62 ± 2.147	47.83 ± 2.069
		AgNPs	67.93 ± 2.939	61.75 ± 2.671	75.47 ± 3.265	68.39 ± 2.959	60.53 ± 2.619	58.35 ± 2.524
		ZnONPs	70.62 ± 3.055	64.2 ± 2.777	78.47 ± 3.395	71.1 ± 3.076	62.93 ± 2.722	60.66 ± 2.624

**Table 4:** Physical and mechanical properties of treated dyed fabrics with/without polymers.

Fabric	Polymer Conc. (10 g/L)	MNPs (10%)	Physical and Mechanical Properties				
			Tensile Strength (N/mm^2^)	Elongation at a Break (%)	Bending Length (cm)	Crease Recovery Angle (Warp + Weft) (°)	Surface Roughness
Cotton	Blank		11.05 ± 0.478	6.4 ± 0.277	3.5 ± 0.151	185.9 ± 8.042	15.21 ± 0.658
	PVA	Without	12.43 ± 0.538	7.2 ± 0.311	3.9 ± 0.169	209.14 ± 9.047	13.31 ± 0.576
		AgNPs	11.73 ± 0.507	6.8 ± 0.294	3.7 ± 0.16	197.4 ± 8.54	13.81 ± 0.597
		ZnONPs	11.74 ± 0.508	6.8 ± 0.294	3.7 ± 0.16	197.48 ± 8.543	14.11 ± 0.61
	Chitosan	Without	13.54 ± 0.586	7.8 ± 0.337	4.3 ± 0.186	227.73 ± 9.852	18.63 ± 0.806
		AgNPs	12.48 ± 0.54	7.2 ± 0.311	4 ± 0.173	210.01 ± 9.085	12.15 ± 0.526
		ZnONPs	12.59 ± 0.545	7.3 ± 0.316	4 ± 0.173	211.74 ± 9.16	14.97 ± 0.648
	CMC	Without	12.98 ± 0.562	7.5 ± 0.324	4.1 ± 0.177	218.43 ± 9.449	15.97 ± 0.691
		AgNPs	13.3 ± 0.575	7.7 ± 0.333	4.2 ± 0.182	223.75 ± 9.679	13.29 ± 0.575
		ZnONPs	12.96 ± 0.561	7.5 ± 0.324	4.1 ± 0.177	217.97 ± 9.429	14.74 ± 0.638
Polyester	Blank		21 ± 0.908	10.3 ± 0.446	4.4 ± 0.19	204.6 ± 8.851	18.3 ± 0.792
	PVA	Without	23.63 ± 1.022	11.6 ± 0.502	5 ± 0.216	230.18 ± 9.958	20.59 ± 0.891
		AgNPs	22.3 ± 0.965	10.9 ± 0.472	4.7 ± 0.203	217.26 ± 9.399	16.14 ± 0.698
		ZnONPs	22.31 ± 0.965	10.9 ± 0.472	4.7 ± 0.203	217.34 ± 9.402	18.34 ± 0.793
	Chitosan	Without	25.73 ± 1.113	12.6 ± 0.545	5.4 ± 0.234	250.64 ± 10.843	22.42 ± 0.97
		AgNPs	23.72 ± 1.026	11.6 ± 0.502	5 ± 0.216	231.13 ± 9.999	15.99 ± 0.692
		ZnONPs	23.92 ± 1.035	11.7 ± 0.506	5 ± 0.216	233.04 ± 10.081	18.92 ± 0.818
	CMC	Without	24.68 ± 1.068	12.1 ± 0.523	5.2 ± 0.225	240.41 ± 10.4	21.5 ± 0.93
		AgNPs	25.28 ± 1.094	12.4 ± 0.536	5.3 ± 0.229	246.26 ± 10.653	16.66 ± 0.721
		ZnONPs	24.62 ± 1.065	12.1 ± 0.523	5.2 ± 0.225	239.9 ± 10.378	19.03 ± 0.823
Cotton/Polyester	Blank		23.2 ± 1.004	13.3 ± 0.575	3.7 ± 0.16	200 ± 8.652	15.5 ± 0.671
	PVA	Without	26.1 ± 1.129	15 ± 0.649	4.2 ± 0.182	225 ± 9.734	17.44 ± 0.754
		AgNPs	24.64 ± 1.066	14.1 ± 0.61	3.9 ± 0.169	212.38 ± 9.188	13.67 ± 0.591
		ZnONPs	24.65 ± 1.066	14.1 ± 0.61	3.9 ± 0.169	212.46 ± 9.191	15.54 ± 0.672
	Chitosan	Without	28.42 ± 1.229	16.3 ± 0.705	4.5 ± 0.195	245 ± 10.599	18.99 ± 0.822
		AgNPs	26.21 ± 1.134	15 ± 0.649	4.2 ± 0.182	225.93 ± 9.774	13.54 ± 0.586
		ZnONPs	26.42 ± 1.143	15.1 ± 0.653	4.2 ± 0.182	227.8 ± 9.855	16.02 ± 0.693
	CMC	Without	27.26 ± 1.179	15.6 ± 0.675	4.3 ± 0.186	235 ± 10.166	18.21 ± 0.788
		AgNPs	27.92 ± 1.208	16 ± 0.692	4.5 ± 0.195	240.72 ± 10.414	14.11 ± 0.61
		ZnONPs	27.2 ± 1.177	15.6 ± 0.675	4.3 ± 0.186	234.51 ± 10.145	16.11 ± 0.697

## Data Availability

All required data is in the text and there is no other data missed.

## Author Declarations

The authors declare that the data supporting the findings of this study are available in the article.
